# Supplementary material for: Platinum retention in plasma, urine, and normal colonic mucosa in cisplatin-treated testicular cancer survivors
Source: PLoS One. 2024 Nov 14;19(11):e0312994. doi: 10.1371/journal.pone.0312994 (PMC11563357; doi:10.1371/journal.pone.0312994)
Supplement: S1 File — (DOCX) [file pone.0312994.s001.docx]

**Supplementary Table 1. Association between number of cycles of cisplatin and years since treatment and the log-transformed platinum concentration in plasma.**

|  | Estimate | Std. Error | t-value | p-value |
| --- | --- | --- | --- | --- |
| Intercept | 3.76291 | 0.09523 | 39.514 | <0.0001 |
| 350-450 mg/m^2^ cisplatin | 0.35222 | 0.13014 | 2.706 | 0.0078 |
| >450 mg/m^2^ cisplatin | 0.54702 | 0.19029 | 2.875 | 0.0048 |
| ≥20-29 years since treatment | -0.79798 | 0.12995 | -6.141 | <0.0001 |
| ≥30 years since treatment | -1.45877 | 0.17166 | -8.498 | <0.0001 |

**Supplementary Table 2. Association between number of cycles of cisplatin and years since treatment and the platinum concentration in urine.**

|  | Estimate | Std. Error | t-value | p-value |
| --- | --- | --- | --- | --- |
| REF | 6.1788 | 0.1758 | 35.141 | <0.0001 |
| 350-450 mg/m^2^ cisplatin | 0.3595 | 0.2379 | 1.511 | 0.13 |
| >450 mg/m^2^ cisplatin | 0.8087 | 0.3459 | 2.338 | 0.021 |
| ≥20-29 years since treatment | -0.9684 | 0.2274 | -4.259 | <0.0001 |
| ≥30 years since treatment | -1.3722 | 0.3068 | -4.473 | <0.0001 |

**Supplementary Table 3. Correlation between number of cycles of cisplatin and years since treatment and the platinum concentration in normal colonic mucosa.**

|  | Estimate | Std. Error | t-value | p-value |
| --- | --- | --- | --- | --- |
| REF | 0.20838 | 0.25376 | 0.821 | 0.42 |
| 350-450 mg/m^2^ cisplatin | -0.73198 | 0.37859 | -1.933 | 0.059 |
| >450 mg/m^2^ cisplatin | 0.06966 | 0.48949 | 0.142 | 0.89 |
| ≥20-29 years since treatment | -1.11801 | 0.43438 | -2.574 | 0.013 |
| ≥30 years since treatment | 0.26350 | 0.50283 | 0.524 | 0.60 |
