# Supplementary figures and images for: Platinum retention in plasma, urine, and normal colonic mucosa in cisplatin-treated testicular cancer survivors
Source: PLoS One. 2024 Nov 14;19(11):e0312994. doi: 10.1371/journal.pone.0312994 (PMC11563357; doi:10.1371/journal.pone.0312994)

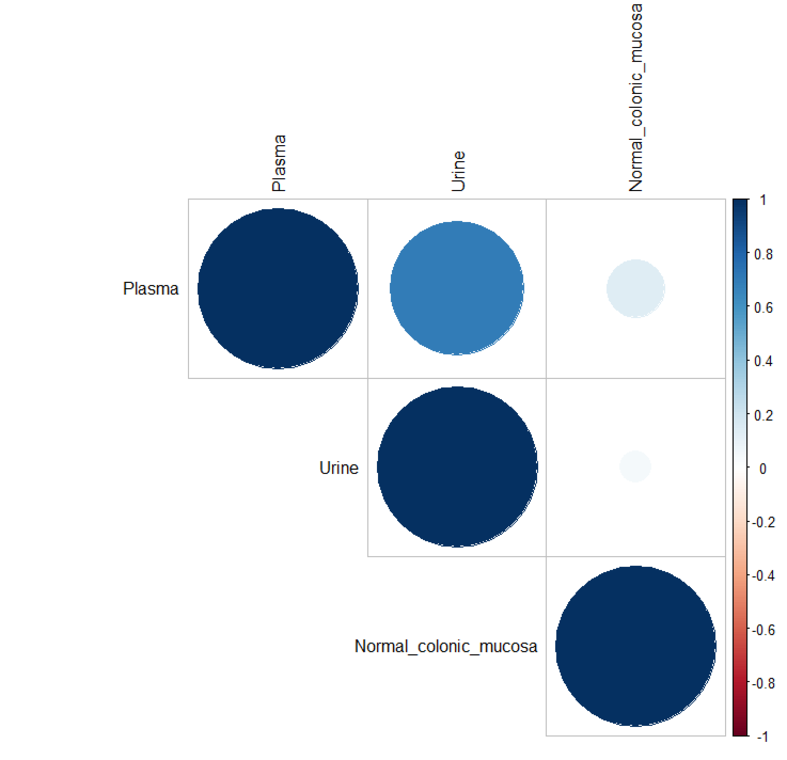

Supplement: S1 Fig — (TIF) [file pone.0312994.s002.tif]

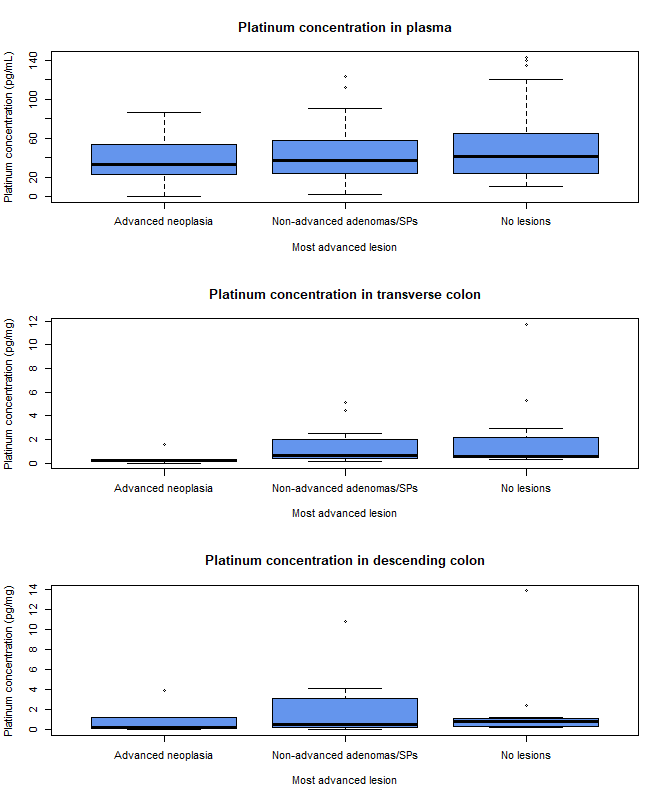

Supplement: S2 Fig — (TIFF) [file pone.0312994.s003.tiff]
